# Oppositional COMT Val158Met effects on resting state functional connectivity in adolescents and adults

**DOI:** 10.1007/s00429-014-0895-5

**Published:** 2014-10-16

**Authors:** Bernhard M. Meyer, Julia Huemer, Ulrich Rabl, Roland N. Boubela, Klaudius Kalcher, Andreas Berger, Tobias Banaschewski, Gareth Barker, Arun Bokde, Christian Büchel, Patricia Conrod, Sylvane Desrivières, Herta Flor, Vincent Frouin, Jurgen Gallinat, Hugh Garavan, Andreas Heinz, Bernd Ittermann, Tianye Jia, Mark Lathrop, Jean-Luc Martinot, Frauke Nees, Marcella Rietschel, Michael N. Smolka, Lucie Bartova, Ana Popovic, Christian Scharinger, Harald H. Sitte, Hans Steiner, Max H. Friedrich, Siegfried Kasper, Thomas Perkmann, Nicole Praschak-Rieder, Helmuth Haslacher, Harald Esterbauer, Ewald Moser, Gunter Schumann, Lukas Pezawas

**Affiliations:** 1 Department of Psychiatry and Psychotherapy, Medical University of Vienna, Währinger Gürtel 18-20, 1090 Vienna, Austria; 2 Department of Child and Adolescent Psychiatry, Medical University of Vienna, Vienna, Austria; 3 MR Centre of Excellence, Center for Medical Physics and Biomedical Engineering, Medical University of Vienna, Vienna, Austria; 4 Central Institute of Mental Health, Faculty of Clinical Medicine Mannheim, Heidelberg University, Mannheim, Germany; 5 Institute of Psychiatry, King’s College, London, UK; 6 Institute of Neuroscience, Trinity College, Dublin, Ireland; 7 University Medical Centre Hamburg-Eppendorf, Hamburg, Germany; 8 Department of Psychiatry, Université de Montreal, CHU St. Justine Hospital, Montreal, Canada; 9 Neurospin, Commissariat à l’Energie Atomique, CEA-Saclay Center, Paris, France; 10 Department of Psychiatry and Psychotherapy, Campus Charité Mitte, Berlin, Germany; 11 Department of Psychiatry and Psychology, University of Vermont, Burlington, USA; 12 Physikalisch-Technische Bundesanstalt, Berlin, Germany; 13 McGill University and Génome Québec Innovation Centre, Montreal, Canada; 14 Institut National de la Santé et de la Recherche Médicale, INSERM CEA Unit 1000 “Imaging and Psychiatry”, Orsay, France; 15 Department of Psychiatry and Neuroimaging Center, Technische Universität Dresden, Dresden, Germany; 16 Center for Biomolecular Medicine and Pharmacology, Medical University of Vienna, Vienna, Austria; 17 Department of Psychiatry and Behavioral Sciences, Division of Child and Adolescent Psychiatry and Child Development, Stanford University School of Medicine, Stanford, USA; 18 Department of Laboratory Medicine, Medical University of Vienna, Vienna, Austria

**Keywords:** Catechol-*O*-methyltransferase, Dopamine, Adolescents, Cognition, Functional neuroimaging, Magnetic resonance imaging

## Abstract

Prefrontal dopamine levels are relatively increased in adolescence compared to adulthood. Genetic variation of *COMT* (COMT Val158Met) results in lower enzymatic activity and higher dopamine availability in Met carriers. Given the dramatic changes of synaptic dopamine during adolescence, it has been suggested that effects of COMT Val158Met genotypes might have oppositional effects in adolescents and adults. The present study aims to identify such oppositional COMT Val158Met effects in adolescents and adults in prefrontal brain networks at rest. Resting state functional connectivity data were collected from cross-sectional and multicenter study sites involving 106 healthy young adults (mean age 24 ± 2.6 years), gender matched to 106 randomly chosen 14-year-olds. We selected the anterior medial prefrontal cortex (amPFC) as seed due to its important role as nexus of the executive control and default mode network. We observed a significant age-dependent reversal of COMT Val158Met effects on resting state functional connectivity between amPFC and ventrolateral as well as dorsolateral prefrontal cortex, and parahippocampal gyrus. Val homozygous adults exhibited increased and adolescents decreased connectivity compared to Met homozygotes for all reported regions. Network analyses underscored the importance of the parahippocampal gyrus as mediator of observed effects. Results of this study demonstrate that adolescent and adult resting state networks are dose-dependently and diametrically affected by COMT genotypes following a hypothetical model of dopamine function that follows an inverted U-shaped curve. This study might provide cues for the understanding of disease onset or dopaminergic treatment mechanisms in major neuropsychiatric disorders such as schizophrenia and attention deficit hyperactivity disorder.

**Electronic supplementary material:**

The online version of this article (doi:10.1007/s00429-014-0895-5) contains supplementary material, which is available to authorized users.

## Introduction

Human dopaminergic signaling is critically modulated by a variety of alterations occurring during adolescence such as increases of basal dopamine (DA) levels and changes of DA turnover resulting in a peak of prefrontal dopaminergic neurotransmission in early adolescence that declines thereafter (Andersen et al. 1997; Rosenberg and Lewis 1994, 1995; Teicher et al. 1993; Wahlstrom et al. 2010). Moreover, prefrontal dopaminergic innervation comes to a climax during adolescence and it continuously decreases during adulthood (Rosenberg and Lewis 1995; Tarazi et al. 1999).

DA-mediated behavioral effects have been proposed to follow an inverted U-shaped dose–response curve by some authors (Arnsten 1997; Cools and D’Esposito 2011), with both deficient and excessive amounts of DA activity predicting poor cognitive task performance. Due to lacking prefrontal cortical DA transporters (Sesack et al. 1998), DA availability in the PFC is critically dependent on its degrading enzyme catechol-*O*-methyltransferase (COMT) (Yavich et al. 2007). Its function is known to be affected by a functional single nucleotide polymorphism (SNP) in *COMT* (G-to-A base-pair substitution) leading to a methionine (Met) valine (Val) substitution at codons 108/158 (COMT Val158Met). Carriers of the Met allele have been found to display a fourfold decrease in enzymatic activity compared to Val allele carriers going along with an increase of prefrontal DA activity (Lachman et al. 1996; Lotta et al. 1995). Under physiological conditions individuals homozygous for the Met allele are thought to be placed near the apex of the inverted U-shaped curve, whereas Val allele carriers reside more at the lower end of the curve due to the Val allele’s increased DA metabolism rate. The specific position of COMT genotypes on the hypothetical inverted U-shaped curve has been demonstrated to change, when synaptic dopamine is pharmacologically increased leading to a shift to the right along the curve (Apud et al. 2007; Goldman-Rakic et al. 2000; Mattay et al. 2003).

Similar changes are likely to arise also during adolescence due to physiologically increased dopamine levels compared to adulthood. Hence, the “optimal genotype” for prefrontal functioning might differ between adolescents and adults which could be explained by a transposition along the hypothetical inverted U-shaped curve (Apud et al. 2007; Wahlstrom et al. 2010), a notion supported by several pharmacological, imaging, or behavioral reports (Apud et al. 2007; Gothelf et al. 2005, 2013; Mattay et al. 2003).

A vast body of the literature has highlighted the importance of COMT Val158Met with respect to PFC activation and engagement of prefrontal networks during cognitively demanding tasks in adults (Mier et al. 2010; Tunbridge et al. 2013). Only recently, scientists shifted their focus towards the specific role of DA and *COMT* genotypes on default mode network (DMN) and executive control network (ECN) function during rest (Beckmann et al. 2005; Cole et al. 2013; Dang et al. 2012; Delvaux et al. 2013; Lee et al. 2011; Liu et al. 2010; Minzenberg et al. 2011; Tian et al. 2013; Tunbridge et al. 2013).

While imaging studies have shown COMT Val158Met effects on prefrontal brain networks during rest in adults, knowledge on its impact during brain development is still sparse. This is remarkable, given that the “functional connectome” provides an attractive quantitative phenotype for developmental changes of the brain’s intrinsic architecture (Biswal et al. 2010) and an interesting biomarker for translational psychiatric research (Smucny et al. 2014). Hence, we conducted a functional magnetic resonance imaging (fMRI) study investigating COMT Val158Met effects on prefrontal coupling at rest in a large sample of healthy adolescents and adults. The anterior medial PFC (amPFC) has been chosen as seed for our functional connectivity analyses, because it represents the most prominent intersection of the DMN and ECN within the PFC (Beckmann et al. 2005; Smith et al. 2009). Based on above-mentioned previous reports highlighting striking differences in DA signaling between adolescents and adults, we hypothesized that COMT Val158Met leads to oppositional prefrontal functional coupling in both developmental groups.

## Materials and methods

### Subjects

Cross-sectional data were collected from a single-center study site involving 106 healthy young adults (M/F = 49/57, Val/Val *n* = 24, Val/Met *n* = 59, Met/Met *n* = 23), gender- and genotype-matched to 106 randomly chosen 14-year-olds (M/F = 49/57, Val/Val *n* = 24, Val/Met *n* = 59, Met/Met *n* = 23) from multiple-center study sites. Distribution of genotypes did not significantly deviate from the Hardy–Weinberg equilibrium (*p* = 0.33). The study was performed in accordance with the Declaration of Helsinki. Local ethics committees approved all study procedures. Subjects at all sites underwent a clinical interview for DSM-IV Axis I disorders [Structured Clinical Interview for DSM Disorders (SCID), Development and Well-Being Assessment (DAWBA)] and a thorough physical examination. Only adult or adolescent subjects without any psychiatric lifetime diagnosis, clinically significant abnormalities and current or previous substance abuse except nicotine dependence were enrolled in the study.

#### Adolescent sample

Resting state neuroimaging data and COMT genotype data were retrieved from the European-Commission funded “IMAGEN study” sample, which encompassed exclusively 14-year-old adolescents (Schumann et al. 2010). There was no direct financial benefit from participation in this study. Yet, participants received financial compensation for their expenditure of time, and travel expenses. Written informed assent and consent were obtained, respectively, from all adolescents and their parents after complete description of the study. A precise description of recruitment and assessment procedures, and exclusion and inclusion criteria was published previously (Schumann et al. 2010).

#### Adult sample

The adult sample (age range 18–33 years; mean age 24 ± 2.6 years) was retrieved from the “Viennese Imaging Genetics Project” funded by the Austrian Science Fund (FWF), the Austrian National Bank (OENB), and the Institute for the Study of Affective Neuroscience (ISAN). All adult participants were recruited by online advertisements, announcements on bulletin boards and word of mouth at the Medical University of Vienna, Austria. Participants were financially compensated for their expenditure of time.

### Genotyping

#### Adolescent sample

A precise description of genotyping procedures has been published previously (Schumann et al. 2010).

#### Adult sample

Genotyping was performed at the Department of Laboratory Medicine, Medical University of Vienna, Austria. DNA was isolated from EDTA blood samples using the Magna Pure LC DNA Isolation Kit (Roche). COMT Val158Met genotyping was performed by means of a tetra-primer amplification refractory mutation system-polymerase chain reaction (ARMS-PCR), according to a previously published protocol (Ruiz-Sanz et al. 2007).

### Magnetic resonance imaging

All participants were instructed to close their eyes, stay awake and keep as immobile as possible during resting state image acquisition.

#### Adolescent sample

Structural and functional magnetic resonance imaging data used in this multicenter study were obtained using 3T Siemens MRI scanners at three study sites. For each sequence, a set of parameters compatible with all scanners, particularly those directly affecting image contrast or signal-to-noise, was devised and held constant across sites to minimize differences between scanners (Schumann et al. 2010). Further details on functional and structural data acquisition are described elsewhere (Schumann et al. 2010).

#### Adult sample

Imaging data were collected at a single study site using a 3T Siemens TIM Trio scanner equipped with a Siemens 12-channel head coil. Head movements were restricted using foam pillows and recorded during functional image acquisition. Structural images were obtained using the 3D MPRAGE sequence (repetition time (TR)/echo delay time (TE) = 2,300/4.21 ms, flip angle = 9°, inversion time = 900 ms, voxel size = 1 × 1 × 1.1 mm). Functional data were acquired via a phase-corrected blipped gradient echo (GE), single shot EPI sequence (TR/TE = 42/2,000 ms, 96 × 96 matrix, 210 mm square FOV, 20 axial slices, slice thickness = 4 mm, slice gap = 1 mm) using an interleaved slice acquisition scheme.

#### Preprocessing

Preprocessing steps were identically performed for all subjects with AFNI (http://afni.nimh.nih.gov/afni/) by applying standard procedures that have been executed within a R software framework (http://cran-r-project.or/) for automation purposes (Boubela et al. 2012). Preprocessing included reconstruction, slice-timing correction, rigid-body motion correction, and alignment to the individual anatomical brain using a 12-point affine transformation. The first five volumes were removed to ensure that magnetization equilibrium was reached. For statistical reasons, the last seven volumes of adolescents’ data were also removed to achieve identical trial length (175 TRs) in both samples. ANATICOR artifact regression analysis was applied to resting state time series to control for nuisance signals and localized transient hardware artifacts (see http://afni.nimh.nih.gov/sscc/hjj/anaticor/) (Jo et al. 2010). Motion parameters have been generated by the alignment procedure, whereas additional nuisance variables have been estimated from eroded white matter (WM) and cerebrospinal fluid (CSF) masks provided by FreeSurfer anatomical segmentation (processed using FreeSurfer software version 5.1.0 (http://surfer.nmr.mgh.harvard.edu/) on a Linux system (Red Hat Enterprise Linux 5, x86_64 architecture) as described elsewhere (Rabl et al. 2014). For temporal filtering a broad frequency band (0.008–0.15 Hz) was used, which has recently been found to yield the highest reliability in resting state fMRI analysis (Braun et al. 2012). Moreover, studies demonstrated that higher frequencies contain meaningful information when proper noise regression is used (Boubela et al. 2013). Eventually, data underwent a spatial Gaussian blur (full width at half maximum (FWHM) = 6 mm) followed by warping to Talairach–Tournoux stereotactic space and final calculation of functional connectivity maps.

#### Data quality control

All structural and functional datasets were reconstructed and visually inspected for major artifacts before and after significant preprocessing steps. Results of FreeSurfer segmentation were screened for errors including visual inspection of the resulting WM and CSF masks to ensure proper nuisance regression with ANATICOR. With respect to motion, we observed a maximum of motion below 1.9 mm translation. Given the absence of widely accepted thresholds, we chose this trade-off between technical considerations and the risk to introduce a sample selection bias, especially with respect to the more active adolescent subsample. We additionally calculated the root mean square of 3D mean translations (${\text{displacement}} = \sqrt {x^{2} + y^{2} + z^{2} }$) as recommended (Jo et al. 2010; Van Dijk et al. 2012). We found no significant main and interaction effect of displacement for the linear model of COMT Val158Met homozygotes × developmental stage (age) with respect to the covariate gender, applied in analogy to our imaging analyses (gender male vs. female: *b* = 0.029, *t*(89) = 0.66, *p* = 0.51; COMT Val158Met Val/Val vs. Met/Met: *b* = 0.024, *t*(89) = 0.39, *p* = 0.70; developmental stage adult vs. adolescent: *b* = −0.0056, *t*(89) = −0.09, *p* = 0.93; COMT Val158Met Val/Val vs. Met/Met × developmental stage adult vs. adolescent: *b* = −0.051, *t*(89) = −0.60, *p* = 0.55). It is noteworthy that the ANATICOR algorithm implemented in our preprocessing pipeline targets frequently observed scanner and especially motion artifacts (Jo et al. 2010; Van Dijk et al. 2012).

### Statistical analysis

#### Functional connectivity

After above-mentioned preprocessing steps functional datasets were utilized to calculate functional connectivity maps. Time series were extracted from a 4 mm spherical seed placed at the amPFC and averaged. Since previous studies have reported that COMT Val158Met affects the functional coupling of the ECN and DMN, we decided to choose the amPFC as a priori seed due to its prominent role as prefrontal intersection (“dorsal nexus”) of the task-positive ECN and DMN, which is typically deactivated during cognitive tasks (Beckmann et al. 2005; Buckner et al. 2008; Sheline et al. 2010; Smith et al. 2009). Seed coordinates were derived from the literature (Fair et al. 2008) and converted from Montreal Neurological Institute (MNI) (*x* = 1, *y* = 54, *z* = 21) to Talairach–Tournoux space (TLRC) (*x* = 0, *y* = 51, *z* = 18) using non-linear registration (Lacadie et al. 2008).

**Fig. 1:**
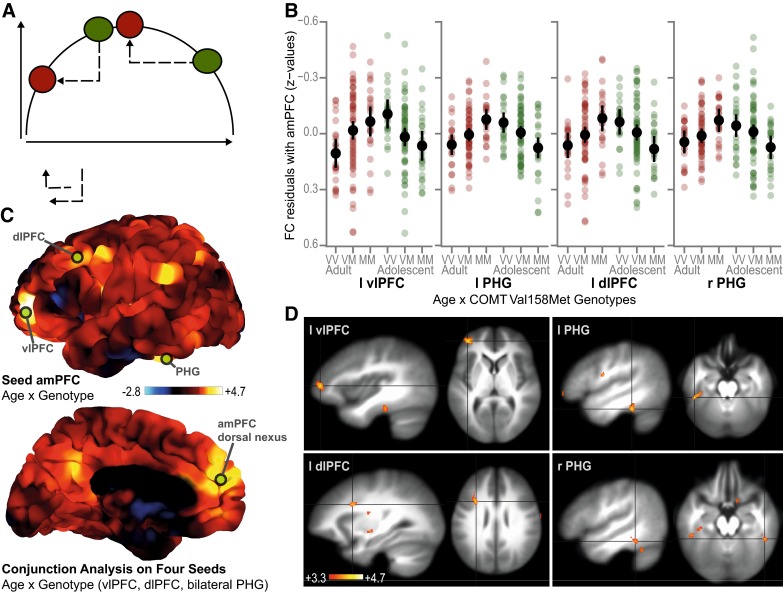
**a** Graph displays working memory performance in dependence of the positioning of COMT Val158Met genotypes along the hypothetical inverted U-shaped curve for adults and adolescents based on previous reports. **b** Graph displays resting state functional connectivity for COMT Val158Met genotypes (*black bars* mean and 95 % CI) in adolescents and adults between amPFC and peak regions (left vlPFC, left PHG, left dlPFC, right PHG), controlled for main effects. Please note the similarity between assumptions on behavioral level (**a**) and resting state functional connectivity data (**b**). **c** Interaction effect of COMT Val158Met × developmental stage (age) controlled for gender. Positive effects indicate a stronger coupling with the seed region for adult Val homozygotes and a weaker coupling for adolescent Val homozygotes compared to Met homozygotes. Results of the seed in the anterior medial prefrontal cortex (amPFC) are shown on the lateral view. Results shown on the medial view are the vertex-wise smallest interaction effect of four lateral seeds to illustrate the extend of the “dorsal nexus” within the DMN. **d** Centered peak coordinates of significant clusters in the left vlPFC, the left PHG, the left dlPFC and the right PHG (*p* < 0.05 corrected). Results were mapped on an averaged anatomical template with a threshold of *p* < 0.001 in line with the family-wise multiple comparison correction (volumetric view, *z*-values). *amPFC* anterior medial prefrontal cortex, *vlPFC* ventrolateral prefrontal cortex, *dlPFC* dorsolateral prefrontal cortex, *PHG* parahippocampal gyrus, *DMN* default mode network

**Fig. 2:**
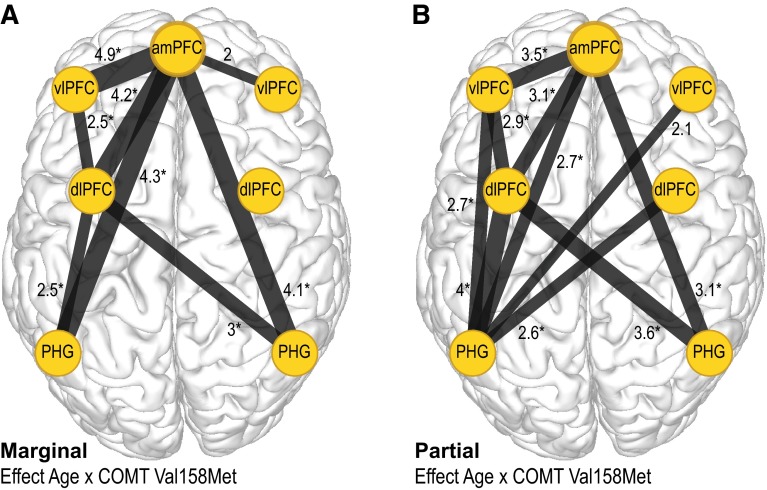
**a** Network representation visualizes marginal (simple Pearson) correlations between peak regions of significant clusters from resting state functional connectivity analyses assessing differences between COMT Val158Met effects in adolescents and adults. As straightforward network translation of the seed-based functional connectivity results, these effects are not necessarily driven by direct connections between each pair of regions. **b** Network representation visualizes partial correlations subserving as estimator for the “true network”. The remaining effect for most connections indicates that oppositional COMT Val158Met network findings in adolescents and adults are robust and not driven by a subset of regions. It is noteworthy, however, that the connection to the left PHG is pronounced when focusing on direct connections in this post hoc analysis (threshold *p* < 0.05, *p < 0.05 FDR corrected, line thickness and numbers indicate *z*-values of the interaction effect). This suggests a relative prominent role of the PHG in the context of this study

Second-level statistics were calculated from single subject Fisher *z*-transformed connectivity maps and tested for a putative interaction between two factors with two levels (homozygous genotype: Val/Val, Met/Met; developmental stage: adolescent, adult) and gender as covariate of no interest within a general linear model (GLM) using AFNI (3dttest++). Initially, we calculated the full model to detect any influence of potential confounders such as study site or motion, which were later removed in favor of a more parsimonious model (Figure S3). Statistical results were not affected when either a linear or a quadratic displacement covariate (Satterthwaite et al. 2012; Van Dijk et al. 2012) was introduced. In addition to our main model (Figs. 1, 2), chosen due to its minimal a priori assumptions, we further provide results of alternative dosing and quadratic models with heterozygotes included in the supplement of this manuscript (Figure S3). First, the dosing model (Figure S3 B) tests an equidistant linear relationship of functional connectivity and the number of Val alleles in interaction with the factor developmental stage (genotype: Val/Val, Val/Met, Met/Met; developmental stage: adolescent, adult). Second, the quadratic model (Figure S3 C) shapes a perfect U-shaped relationship with all six subgroups of COMT and developmental stage ordered from theoretically lowest (Val/Val adult) to highest dopamine levels (Met/Met adolescent) as displayed in Fig. 1a. Even though our results remain robust across these models we focus our report on the parsimonious model with two factors (homozygous genotype, developmental stage) and two levels to avoid inappropriately strict assumptions of equally distant effects in between genotype subgroups. Cluster-wise correction for multiple comparisons was applied using Monte Carlo simulations (3dClustSim, 10,000 iterations, smoothness estimation with 3dFWHMx, dimensions: 74 × 87 × 69 grid, 2.19 × 2.19 × 2.19 mm^3^, a minimum cluster size of 42 voxels yielded a corrected *p* value of 0.05) at a rather conservative initial voxel-wise threshold of *p* < 0.001. All corrected cluster *p* values < 0.05 were considered significant.

#### Post hoc analyses

To determine whether significant genotype-developmental stage interaction effects are driven by a limited set of regions, we applied two functional connectivity measures, marginal and partial correlations (Marrelec et al. 2006). At first, we extracted averaged and normalized time series from 4 mm spheres centered at the interaction effect peak for all significant clusters (left ventrolateral prefrontal cortex (vlPFC) −36, 46, 8, left dorsolateral prefrontal cortex (dlPFC) −30, 11, 28, left parahippocampal gyrus (PHG) −47, −35, −18, right PHG 49, −39, −14). Additionally, we added the contralateral counterparts (right vlPFC 36, 46, 8, right dlPFC 30, 11, 28) and the a priori chosen seed region (amPFC 0, 51, 18) resulting in a graph with seven nodes.

Marginal correlation analysis refers to a simple Pearson correlation between averaged and normalized seed-based time series. This full correlation approach is the most intuitive and a widely used association measure for direct and also indirect network connection analyses (Smith et al. 2011). Moreover, it is a straightforward translation of the present functional connectivity analyses into a network representation and subserves as reference for the subsequent “true network analysis”. We further used partial correlations to estimate the “true network” of direct connections between chosen regions of interest (ROIs). Thereby, we correlated each pair of normalized time series while additionally regressing out each other ROI’s time series (Marrelec et al. 2006). This method provides no directionality information, but is considered a valid data-driven surrogate for effective connectivity approaches due to its ability to remove indirect connections and strengthen direct connections (Smith et al. 2010). Both methods are model-free network integration estimators as they do not require a priori assumptions in contrast to effective connectivity approaches like dynamic causal modeling (DCM) (Kasess et al. 2010). First-level marginal and partial Fisher *z*-transformed correlation matrices were calculated using AFNI (@ROI_Corr_Mat). Second-level statistics (significance level: *p* < 0.05) were calculated in analogy to the mentioned procedures for the present a priori approach. In addition, a conjunctional analysis has been performed comprising seeds in the vlPFC, dlPFC and the bilateral PHG which aims to provide a statistically conservative post hoc overview of genotype × developmental stage effects, with each surface-vertex representing the smallest interaction effect for these four seeds. It is noteworthy that results of multiple comparison correction (*p < 0.05 FDR, false discovery rate) for the network analysis (Fig. 2) and the significance values of the conjunction analysis (Fig. 1c) are less informative due to the post hoc nature and circularity issues inherent in these calculations (Kriegeskorte et al. 2009).

### Creation of figures and tables

Statistical volumetric results were displayed with AFNI on an average anatomical brain of all subjects included in this study. Surface cardinal views were generated by mapping average volumetric statistics on default pial surfaces provided by SUMA software (http://afni.nimh.nih.gov/afni/suma/). Complementary statistics and plots were prepared with R 3.1.1. Plots of extracted functional connectivity peak values (2 mm spherical average) represent residual values after controlling for gender, developmental stage, and genotype. To illustrate study site homogeneity, all site-specific peak values are displayed in Figure S4. Notably, the inclusion of study site as covariate did not alter the magnitude of the effect described below (Figure S3, S4). We used the R package igraph for graph network and ggplot2 for scatterplot visualizations, and Adobe Illustrator CS5 (vers. 15.0.0) for artwork.

## Results

### Adolescents vs. adults

The analysis of the main developmental stage (age) effect replicates previous reports and is, therefore, reported only briefly (Fair et al. 2008; Kelly et al. 2009). We found significant increases of functional connectivity in adolescents compared to adults (*p* < 0.05 corrected, voxel-wise threshold *p* < 0.001), primarily in DMN regions (Raichle et al. 2001; Scharinger et al. 2014) including the medial PFC, posterior cingulate cortex, anterior temporal lobe, anterior insula, inferior frontal gyrus, hippocampus, thalamus and other subcortical nuclei (Figure S1).

### Functional connectivity: COMT × developmental stage

In accordance with our primary hypothesis of a developmental stage dependent reversal of COMT Val158Met genotype effects (Fig. 1a), we observed significant interaction effects between developmental stage (adolescent vs. adult) and COMT genotype on resting state functional connectivity between amPFC and left vlPFC, left dlPFC, and bilateral PHG after correcting for multiple comparisons (Fig. 1c, d; Table 1). With respect to the directionality of observed effects, we found the Val allele to be associated with increases of functional coupling in a dose-dependent manner in adults and vice versa in adolescents for all brain regions showing significant interaction effects (Fig. 1b; Table 1). It is noteworthy that a graphical representation of extracted functional connectivity values follows exactly the hypothetical inverted U-shaped curve model (Fig. 1b).

**Table 1:** Comparison of COMT Val158Met effects on prefrontal functional coupling between adolescents and adults

Region	Size (mm^3^)	*x*	*y*	*z*	*z* value	*p* uncorrected	*p* corrected
Left vlPFC	1,205	36	−46	8	4.66	<0.0001	<0.001
Left PHG	765	47	35	−18	4.59	<0.0001	<0.005
Left dlPFC	534	30	−11	28	4.36	<0.0001	<0.05
Right PHG	492	−49	39	−14	4.04	<0.0001	<0.05

*vlPFC* ventrolateral prefrontal cortex, *PHG* parahippocampal gyrus, *dlPFC* dorsolateral prefrontal cortex, *x*, *y*, *z* coordinates in Talairach space, *p corrected* cluster-corrected *p* values

Furthermore, we analyzed post hoc the investigated adolescent and adult sample separately to calculate genotype effects within each developmental stage and study site independently. In line with our results of the combined adolescent and adult sample (Fig. 1), we found opposite directions of COMT Val158Met effects in adolescents and adults indicating that our combined analysis was not biased by a specific study site. It is noteworthy that genotype-related differences in functional connectivity have been more pronounced in adolescents compared to adults within all regions showing significant interaction effects (Figure S2 D, F) as well as most other brain regions in a global manner in this separate analysis (Figure S2 C, E). Other supplemental calculations addressing alternative statistical models such as the employment of more rigid a priori assumptions (Figure S3) or effects of a potential bias of study site (Figure S3, Figure S4) can be found in detail in the supplement of the manuscript. All results indicate the validity of conclusions being drawn in the combined analysis described above.

### Network analysis: COMT × developmental stage

To further investigate the network of brain regions showing significant interaction effects (vlPFC, dlPFC, PHG) as well as the chosen seed region (amPFC), we applied marginal and partial correlation analyses to the combined sample of adolescents and adults (Marrelec et al. 2006). Marginal correlation analyses revealed significant interaction effects between genotype and developmental stage with respect to functional connectivity between left vlPFC and dlPFC as well as between left dlPFC and bilateral PHG, in addition to the network representation of results reported above (amPFC-vlPFC, amPFC-dlPFC, amPFC-PHG) (Fig. 2a). Partial correlation analyses representing estimates of the “true network” and direct connections showed that all seed-based functional connectivity results reported above were still present and statistically robust (amPFC-vlPFC, amPFC-dlPFC, amPFC-PHG) (Fig. 2b). Additionally, partial correlation analyses emphasized the centrality of the left PHG compared to marginal correlations due to the increased number of significant connections with this region (Fig. 2).

## Discussion

The present study investigating resting state connectivity in healthy adolescents and adults has identified oppositional genotype effects of COMT Val158Met within a neural network encompassing amPFC, vlPFC, dlPFC, and PHG. Moreover, a simple network analysis highlighted the specific importance of the PHG as mediator of these diametrical genotype effects in adolescents and adults. With respect to directionality, adult Val homozygotes of COMT Val158Met showed an increased coupling between the amPFC and vlPFC, dlPFC, and PHG compared to adult Met homozygotes, whereas allele dose-dependent opposing effects were detected for adolescents (Fig. 1b).

The choice of the amPFC as seed region for performed functional connectivity analyses was due to its role as prominent nexus between the DMN and ECN (Beckmann et al. 2005; Smith et al. 2009), two systems, which are without doubt under dopaminergic control (Goldman-Rakic et al. 2000; Meyer-Lindenberg et al. 2005). The mentioned regions of interactions (Fig. 1; Table 1) are known to be involved in cognitive control, declarative memory retrieval and encoding (Andrews-Hanna et al. 2014; Smolker et al. 2014).

Overall, our finding of oppositional COMT Val158Met effects in adolescents and adults is in line with previous reports demonstrating a relative DA increase in adolescence compared to adulthood (Andersen et al. 1997; Rosenberg and Lewis 1994, 1995; Teicher et al. 1993). The graphical representation (Fig. 1b) and supplemental statistics (Figure S3) of COMT Val158Met effects in adolescents and adults observed within this study are in accordance with the previously proposed hypothetical dose–response model of DA that follows an inverted U-shaped curve (Floresco and Phillips 2001; Goldman-Rakic et al. 2000; Robbins 2000; Verma and Moghaddam 1996; Williams and Goldman-Rakic 1995; Zahrt et al. 1997) (Fig. 1a). Within this framework, our results would indicate a genotype shift to the left along the inverted U-shaped curve for adolescents relative to the position of adults (Fig. 1a) in analogy to a pharmacologically induced dopamine increase in adults (Apud et al. 2007; Mattay et al. 2003).

A limited number of human and animal studies have investigated the influence of COMT genotype during development (Barnett et al. 2007; Dumontheil et al. 2011; Gothelf et al. 2005, 2013; Lambe et al. 2000; Tunbridge et al. 2007). While the available literature is still inconclusive (Barnett et al. 2007), our findings are in line with a genetically-informed schizophrenia model derived from an orphan disease (Gothelf et al. 2005, 2013). In velo-cardio-facial syndrome (VCFS), a rare disorder with an increased risk of schizophrenia resulting from a microdeletion in 22q11.2, patients are known to lack one copy of *COMT*. In this patient group available evidence suggests that genotype effects of COMT Val158Met on cognitive performance are critically dependent on the maturational stage of the brain (Gothelf et al. 2005). Specifically, it has been suggested that the presence of the Met allele in adolescent VCFS patients combined with age-related DA increases could result in super-optimal DA levels compared to more optimal DA levels found in Val allele carriers in line with our results. This study is further in agreement with results of a study assessing COMT enzyme activity and protein expression along normal PFC maturation that found similar age-dependent changes in the DA system as reported in this manuscript (Tunbridge et al. 2007).

With respect to the impact of *COMT* on the adult “functional connectome” our results can be related to preliminary evidence in adults (Lee et al. 2011; Liu et al. 2010; Sambataro et al. 2009; Tian et al. 2013; Tunbridge et al. 2013). The majority of these studies report a stronger coupling for adult Val carriers in regions engaged during cognitive tasks, which is in line with our result (Lee et al. 2011; Sambataro et al. 2009; Tunbridge et al. 2013). Such an increased functional connectivity may be related to reports of Val-allele-dependent increased cognitive task activation in lateral prefrontal regions that have been interpreted as “inefficient” PFC function likely reflecting suboptimal DA signaling (Egan et al. 2001; Sambataro et al. 2009) and mimicking findings in schizophrenia patients (Callicott et al. 2000; Manoach et al. 1999). Finally, indirect support stems from an electroencephalography study highlighting a Val allele dose-dependent increase in prefrontal functional connectivity (Lee et al. 2011), which is also known to lead to a suppression failure of the DMN (Pomarol-Clotet et al. 2010).

Our network analyses underscore the putative importance of the PHG as central node for observed development-dependent COMT Val158Met effects (Fig. 2). Notably, several studies report on the developmental impact on parahippocampal regions (Grateron et al. 2003; Meyer and Louilot 2014). Interestingly, it has been suggested that a subtle and transient functional blockade during early developmental periods is sufficient to induce schizophrenia-like behavioral and dopaminergic abnormalities in adulthood (Meyer and Louilot 2014; Peterschmitt et al. 2007). While COMT Val158Met is affecting hippocampal-PFC coupling and declarative memory processing (Bertolino et al. 2006; Krach et al. 2010), it is noteworthy that the PHG constitutes the primary hub of the DMN in the medial temporal lobe (Ward et al. 2013) and represents an important input region for the hippocampus (Andrews-Hanna et al. 2014; Squire et al. 2004), which underlines the plausibility of our network analyses and may be related to previous reports of COMT Val158Met effects on hippocampal volume (Honea et al. 2009; Rabl et al. 2014).

While this study provides novel in vivo insights into the effects of brain maturation on DA-related gene effects, it is not without limitations. First of all, the reported changes should not be overstated, particularly as existing data on age-related effects of *COMT* are as yet preliminary (Barnett et al. 2007). Furthermore, *COMT* is well known to be sexually dimorphic due to its steroid-binding site (Tunbridge et al. 2007), which could introduce a gender-related bias (Laatikainen et al. 2013). However, the strict gender-matching algorithm and regression approach applied within this study has likely removed any gender-specific effect of *COMT*. Even though we carefully matched both developmental groups and controlled for main effects, we cannot exclude any potential study site bias. Nonetheless, the observed oppositional genotype effect was qualitatively present in separate analyses of both developmental groups (Figure S2) which supports the validity of our result. Also, with respect to motion parameters, all subjects included have been within an acceptable range as detailed in the methods section. Importantly, no significant effects of motion have been detected for all calculated main and interaction effects. Finally, it needs to be noted that our adult sample was rather young in age, which might limit our conclusions to young adults (Tunbridge et al. 2007).

The present study provides in vivo evidence for opposing COMT Val158Met effects on resting state connectivity in adolescents and adults. This finding emphasizes the notion that psychiatric risk genes encoding for enzymes such as *COMT*, can result in opposite neural outcomes dependent on the age-specific internal availability of their substrate. This underscores the need for future studies that investigate gene effects on a brain systems level along different stages of brain maturation, which might result in a more thorough understanding of mechanisms determining disease onset, clinical symptomatology, and drug response in major psychiatric disorders such as schizophrenia or attention deficit hyperactivity disorder (ADHD).

## Electronic supplementary material

Below is the link to the electronic supplementary material.

Figure S1. Developmental stage (age) main effect related to resting state functional connectivity with the anterior medial prefrontal cortex seed region. Positive values indicate a stronger coupling in the adolescent sample compared to the adult reference group (*z*-values, surface view). (EPS 22655 kb)

Figure S2. Surface view and complementary graph representations (*z*-values). A, B. Interaction effect of COMT Val158Met x developmental stage (age) controlled for gender. Positive effects indicate a stronger coupling with the anterior medial prefrontal cortex (amPFC) for adult Val homozygotes and a weaker coupling for adolescent Val homozygotes compared to Met homozygotes. C-F. Genotype effects for each group, adults and adolescents, analyzed separately, with positive effects indicating a stronger coupling of MM homozygotes. A, C, E. Peak of regions with statistically significant interaction effects are indicated with green circles. (EPS 40749 kb)

Figure S3. Different statistical approaches towards the interaction effect COMT Val158Met x developmental stage (age) with respect to gender. A. VV vs. MM homozygotes according to the main analysis. B. VV, VM, MM dosing effects. C. U-shaped curve quadratic model of the different genotypes and age. D. VV vs. MM, controlled for all study sites. (EPS 11812 kb)

Figure S4. Graph displays resting state functional connectivity (FC) for COMT Val158Met genotypes (black bars – mean and 95% CI) in adolescents and adults between amPFC and peak regions in the left ventrolateral prefrontal cortex (l vlPFC), left parahippocampal gyrus (l PHG), left dorsolateral prefrontal cortex (l dlPFC) and right parahippocampal gyrus (r PHG), controlled for main effects. Each row represents one specific adolescent sample study site. (EPS 3580 kb)
